# Successful repair of traumatic tricuspid regurgitation with concomitant atrial septal perforation and right ventricular pseudoaneurysm: a case report

**DOI:** 10.1186/s44215-025-00211-8

**Published:** 2025-06-03

**Authors:** Kazuki Mori, Takashi Shuto, Takahiro Tashima, Tomoko Fukuda, Naohiko Takahashi, Shinji Miyamoto

**Affiliations:** 1 Department of Cardiovascular Surgery, Oita University, Oita, Japan; 2 Department of Cardiology and Clinical Examination, Oita University, Oita, Japan

**Keywords:** Tricuspid regurgitation, Atrial septal perforation, Right ventricular injury, Blunt chest trauma

## Abstract

**Background:**

Valvular injuries in chest trauma mostly affect the aortic and mitral valves, but traumatic tricuspid regurgitation (TR) remains rare. This report describes the successful repair of traumatic TR secondary to papillary muscle rupture complicated with right ventricular (RV) free wall injury and atrial septal perforation.

**Case presentation:**

A 50-year-old male suffered blunt chest trauma from a tree fall, leading to multiple fractures, mediastinal hematoma, and hemoperitoneum caused by splenic bleeding. Given that heart failure worsened eventually, echocardiography was conducted on day 7, showing significant TR resulting from leaflet prolapse caused by papillary muscle rupture with concomitant 4.8 mm atrial septal perforation and focal RV free wall thinning. Nonetheless, the heart failure was responsive to medical treatment. The patient was then scheduled for surgery 1 month later. The atrial septal defect was closed via direct suture closure. The RV free wall injury presented with scarring and did not require repair. The tricuspid valve repair included suturing the ruptured medial papillary muscle to the RV wall, reconstructing the ruptured posterior leaflet chordae with prosthetic chordae, and securing an annuloplasty ring. Consequently, TR was completely controlled.

**Conclusions:**

Traumatic tricuspid valve injuries are rare. The optimal timing of surgery for traumatic TR remains controversial. However, early diagnosis and intervention are recommended to prevent progressive RV dysfunction and improve the success of tricuspid valve repair.

**Supplementary Information:**

The online version contains supplementary material available at 10.1186/s44215-025-00211-8.

## Background

Traumatic valvular dysfunction is common in the aortic and mitral valves. However, traumatic tricuspid regurgitation (TR) is relatively rare [1]. In the clinical management of traumatic TR, surgery timing, which is influenced by the presence of heart failure and the severity of other traumas, should be carefully considered [2, 3]. Successful valve repair and right ventricular (RV) function preservation require early surgical intervention [4–7]. This case report describes the successful surgical repair of traumatic TR secondary to papillary muscle (PM) rupture complicated with RV free wall injury and atrial septal perforation (ASP).

## Case presentation

**Fig. 1:**
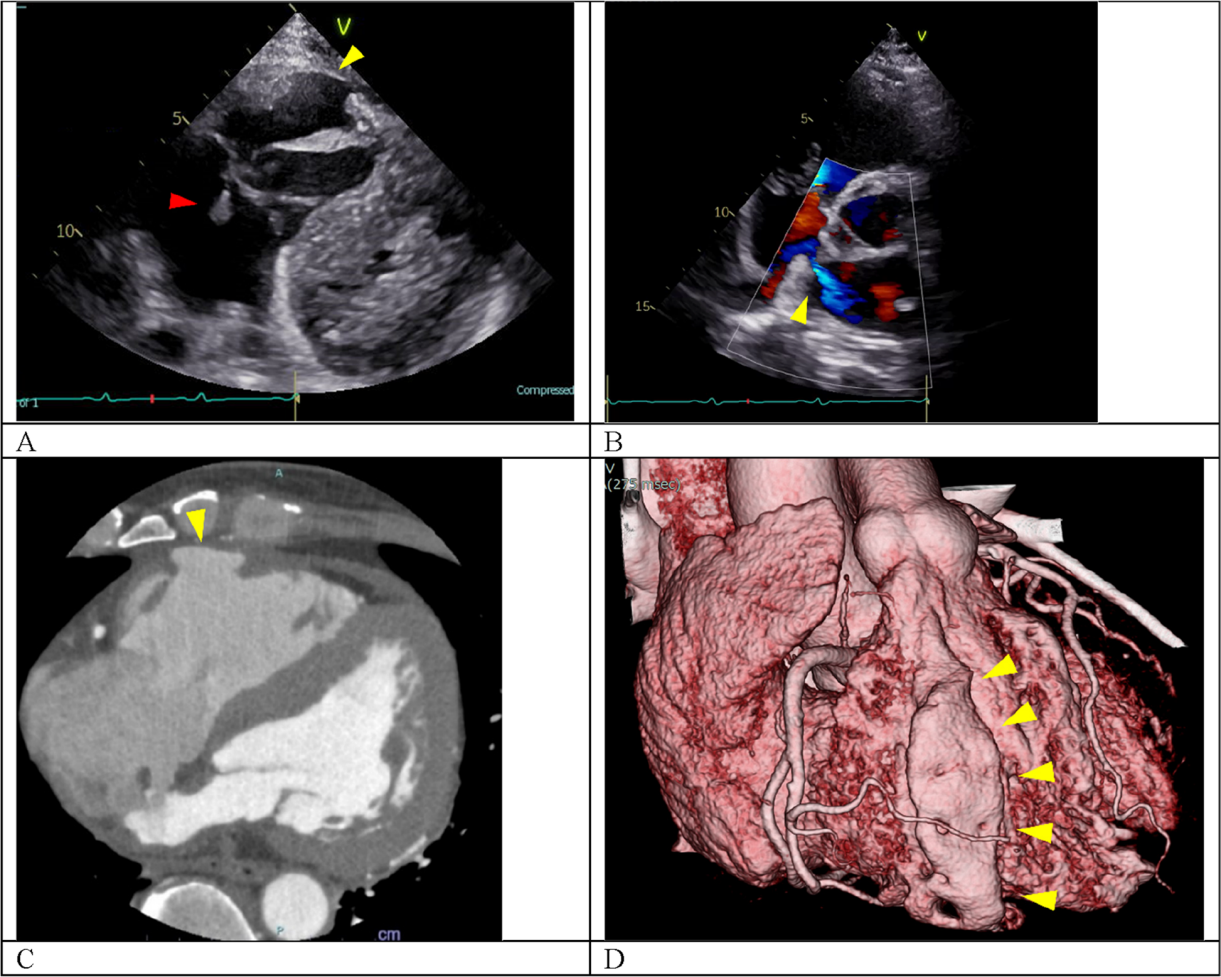
**A** Preoperative echocardiography reveals tricuspid valve prolapse caused by papillary muscle rupture (red arrow), and a thin area of the right ventricular anterior wall (yellow arrow). **B** Color Doppler echocardiography detects a bidirectional shunt across the atrial septum (arrow). **C** Preoperative contrast-enhanced computed tomography shows that the right ventricular anterior wall has a thin region posterior to the sternum (arrow). **D** Three-dimensional computed tomography reconstruction reveals that the right ventricular anterior wall injury corresponds to the sternum (arrows)

A 50-year-old male presented with blunt chest trauma following a tree fall while logging. The patient was transported by a helicopter on an emergency basis, with fluid resuscitation in a preshock state. Multiple traumas, including facial fractures, multiple rib fractures, mediastinal hematoma, hemoperitoneum, and mild pneumothorax, were observed. The intraabdominal bleeding, which resulted from splenic injury, was managed by arterial embolization to achieve hemostasis. On admission to the emergency intensive care unit, the patient developed heart failure worsening. The electrocardiogram revealed a junctional rhythm and a right bundle branch block. Echocardiography on hospitalization day 7 revealed significant TR resulting from leaflet prolapse caused by a ruptured PM (Fig. 1A) and a 4.8-mm atrial septal defect with bidirectional shunting (Supplementary material 1). Furthermore, computed tomography showed focal thinning of the RV free wall adjacent to the sternum (Fig. 1B, C). Compared with the initial findings, the mediastinal hematoma had significantly decreased in size, with no pericardial effusion. Although the patient had significant TR, the heart failure responded favorably to medical treatment. In consideration of the acute posttraumatic state and to minimize the potential for hemorrhage associated with cardiopulmonary bypass, the surgery was scheduled 1 month later.

**Fig. 2:**
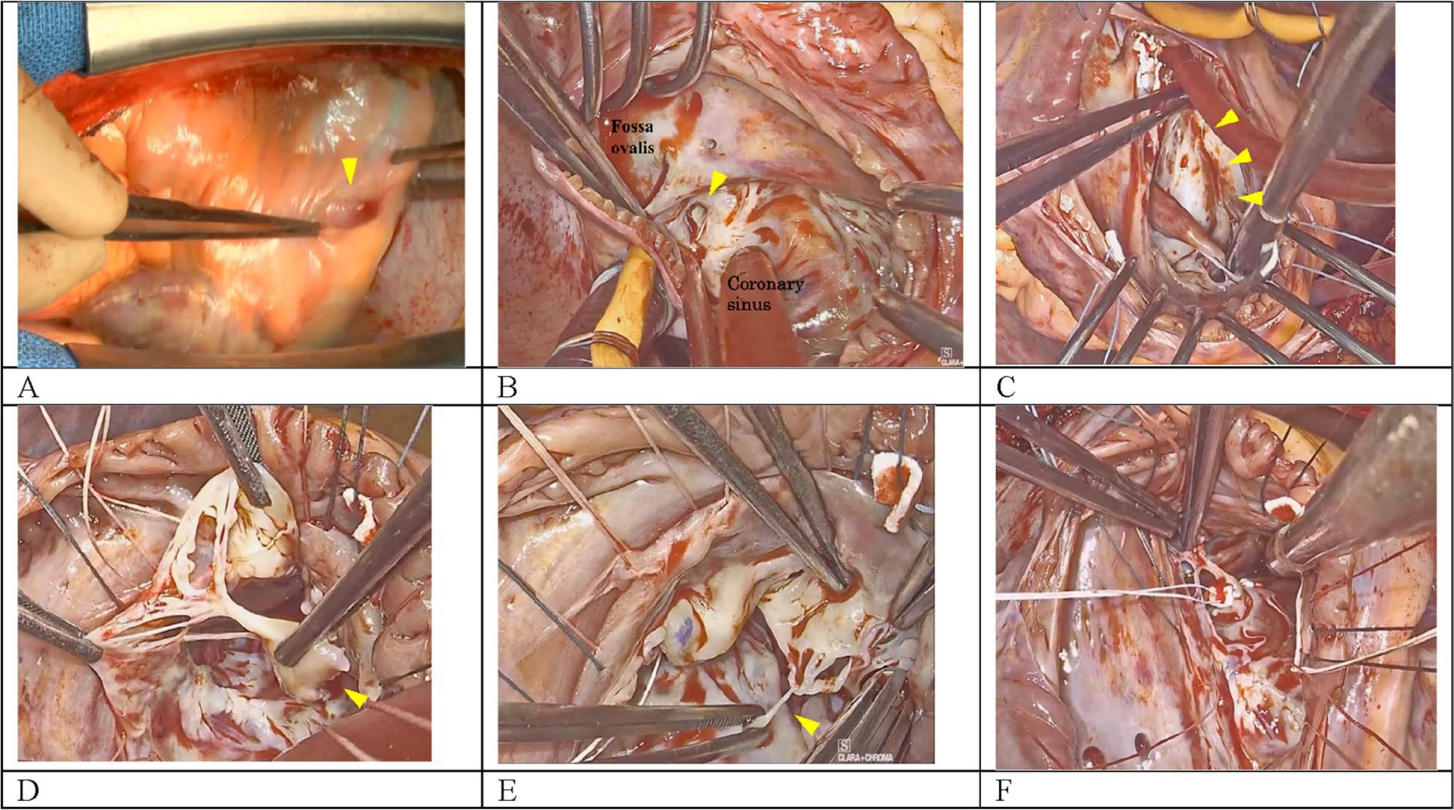
**A** Intraoperative macroscopic findings revealed a hematoma-like appearance (arrow) on the right ventricular anterior surface, but the location of the myocardial injury was unclear. **B** A defect (approx. 5 mm, arrow) was identified within the atrial septum, situated between the fossa ovalis and the coronary sinus. **C** While the right ventricular luminal injury site showed thinning, the internal surface was scarred (arrows). **D** The papillary muscle supporting the chordae tendinea of the anterior leaflet ruptured (arrow). (E) The chordae tendinea supporting the posterior leaflet partially ruptured (arrow). (F) The ruptured papillary muscle was reconstructed by suturing it to the right ventricular septum

**Fig. 3:**
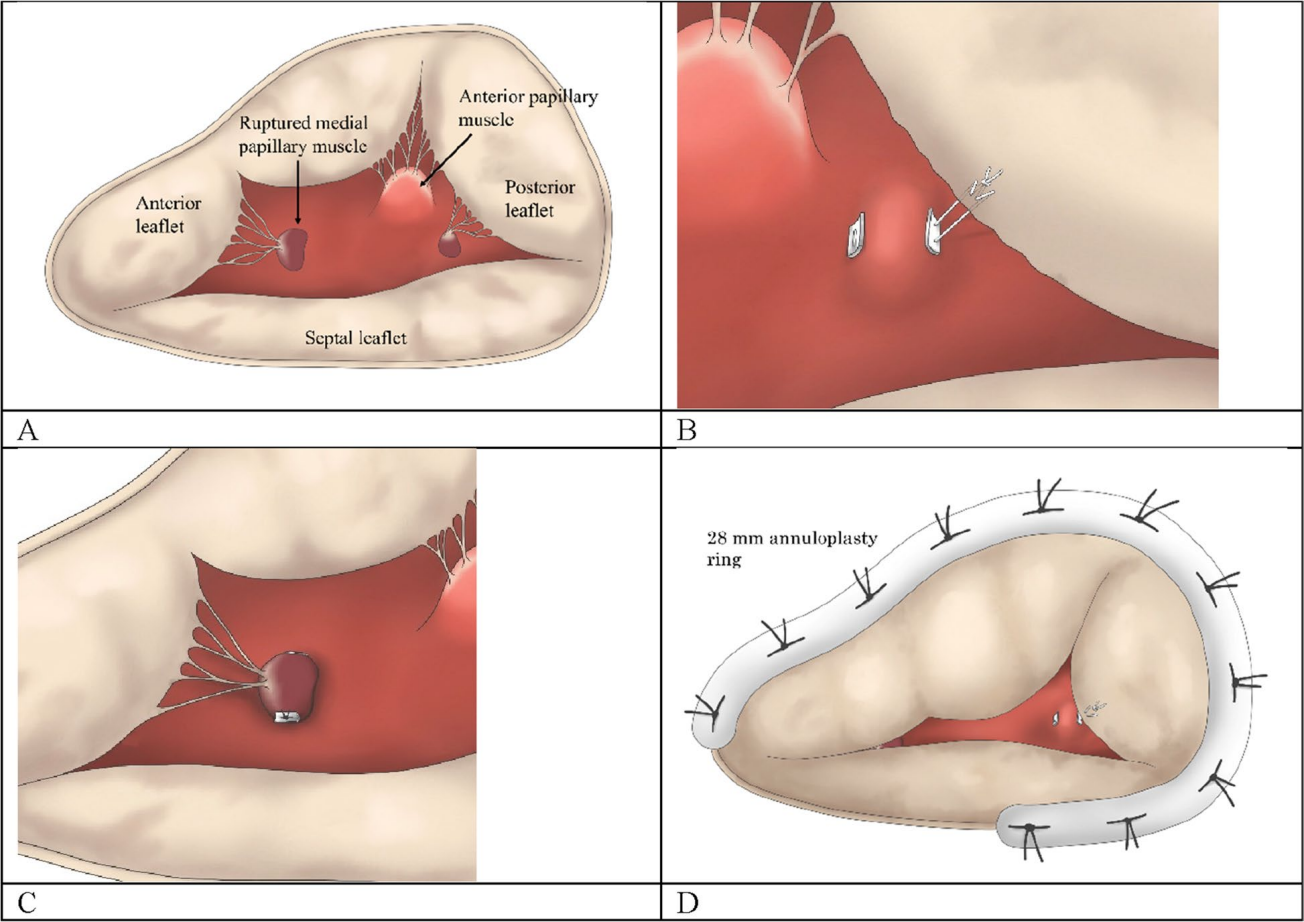
This schematic describes the procedure of tricuspid valve reconstruction. **A** Prolapse of the anterior and posterior leaflets was noted, secondary to rupture of the medial papillary muscle and chordae tendineae of the posterior leaflet. **B** The ruptured chordae tendinea of the posterior leaflet was reconstructed using artificial chordae. **C** The ruptured papillary muscle was reconstructed by suturing it to the right ventricular septum. **D** The reconstruction of the tricuspid valve was completed with the suture of a 28-mm annuloplasty ring

The patient underwent median sternotomy. No adhesions or pericardial effusion were observed. The thinned area of the RV myocardium, as indicated by imaging, could not be visualized (Fig. 2A). Cardiopulmonary bypass was established by cannulating the ascending aorta, superior vena cava, and inferior vena cava. Cardioplegic arrest was achieved by antegrade cardioplegia. After superior and inferior vena cava clamping, right atriotomy was performed. A 5-mm ASP with scarring was observed between the fossa ovalis and the coronary sinus (Fig. 2B) and was closed through direct suture using 4–0 polypropylene mattress sutures supported by pledget felts. The injured RV wall area was thinned but had already scarred (Fig. 2C). The PM, which supported the chordae tendinea of the anterior leaflet, had ruptured (Fig. 2D), as well as the chordae tendinea of the posterior leaflet (Fig. 2E). Meanwhile, the septal leaflet remained intact. The ruptured PM might have been previously attached to the injured RV wall. It was subsequently sutured to the anterior PM with the chordae tendinea of the posterior leaflet using 4–0 polytetrafluoroethylene suture. The ruptured posterior leaflet chordae tendineae was reconstructed at the accessory posterior PM with 4–0 polytetrafluoroethylene suture. Water leakage testing demonstrated anterior leaflet prolapse with significant regurgitation. The ruptured PM was then resutured to the RV septal wall; consequently, the prolapse was resolved (Fig. 2F). A 28-mm Physio Tricuspid annuloplasty ring (Edwards Lifesciences, Irvine, CA, USA) was implanted onto the tricuspid annulus using 3–0 polyester sutures. Figure 3 illustrates a schematic diagram of the tricuspid valve reconstruction. Following water leakage testing, which confirmed no regurgitation, we closed the right atriotomy and weaned the cardiopulmonary bypass. The injured RV free wall was not repaired because of low rupture risk.

The patient received intensive care unit management for 2 days and was discharged on postoperative day 13 without any complications. Follow-up examination at 1 year revealed no TR or atrial septal shunting recurrence. The size of the RV free wall pseudoaneurysm remained unchanged.

## Discussion and conclusions

Valvular injury following chest trauma is more prevalent in the left heart valves than in the right heart valves [8]. Traumatic tricuspid valve injury occurs in only 0.13% of patients with trauma [1], but traumatic TR with concomitant ASP and RV wall injury is even rarer [9].

Traumatic TR is most commonly caused by chordae tendinea rupture, followed by PM rupture and leaflet injury [6, 8], and it affects the anterior leaflet the most [4, 6, 10]. The proposed mechanism of tricuspid valve injury involves the transmission of excessive kinetic energy to the heart, as well as concomitant RV outflow tract obstruction/stenosis and tricuspid valve closure, leading to an acute increase in RV pressure [11]. Subsequently, the valve leaflets, chordae tendinea, and PM exhibited excessive tensile stress, culminating in injury. The PM rupture observed in this case might have resulted from RV free wall injury at the site of PM attachment secondary to direct sternal contact.

Traumatic TR requires an emergency surgical approach in patients with acute heart failure or circulatory insufficiency unresponsive to medical treatment [2]. Without heart failure, early surgical intervention is recommended to reduce RV dysfunction progression [12]. However, the degree of symptom presentation varies from case to case. Many case reports suggest that the diagnosis is often missed in the acute phase because of the absence of symptoms; thus, surgical intervention occurs in the late phase [5–7]. The initial management of our patient also focused on the treatment of other traumatic injuries. The diagnosis was finally made when progressive heart failure became apparent during the patient’s clinical course. Similar cases of intracardiac shunts tended to have severe acute complications [9, 13]. In our patient’s case, the small size of the ASP resulted in a relatively small shunt volume, allowing successful initial medical management. While heart failure may be a sign for the detection, traumatic TR must be considered in the evaluation of high-energy chest trauma, even in asymptomatic patients.

Traumatic valve injuries often affect young, active patients, making valve repair the preferred approach for improving long-term valve function [4–7]. Valve reconstruction requires selecting various techniques, including edge-to-edge repair, chordal transfer, annuloplasty, and the double orifice technique, depending on the degree of the valve lesion [3–5, 10, 14]. We inferred that the ruptured PM, which supported the chordae tendinea of the anterior leaflet, was attached to the RV injury site. However, reattachment to the original site was inappropriate because of RV enlargement caused by the cardiac contusion. Successful reconstruction was achieved by suturing the ruptured PM to the ventricular septum. While some studies documented favorable outcomes in traumatic TR repair, others highlighted the complexity of the procedure [4, 5], citing a 50%–61% rate of need for valve replacement [6, 7]. Prolonging the disease leads to secondary degeneration events, such as annular dilatation, chordal elongation, and leaflet deformation [5, 6]. Therefore, early diagnosis and treatment intervention can result in valve repair success [4–7].

Literature on traumatic cardiac injury extensively covers myocardial rupture, pericardial rupture, intracardiac conduction abnormalities, and ventricular septal dissection. The RV myocardium, which is located anteriorly and has a thin wall, is most susceptible to damage in cardiac trauma [15]. RV pseudoaneurysm management may be controversial. Although RV rupture can lead to a life-threatening condition [16], the present case did not exhibit pseudoaneurysm enlargement or pericardial effusion from the time of injury. Therefore, acute therapeutic intervention was not performed. One month after the injury, the RV injury site was indistinguishable from the epicardial surface. According to the endocardial observation, the lesion had already scarred and transformed into robust tissue. Considering the RV pressure, we believed that the risk of rupture was low, and decided not to repair. Nevertheless, the patient must be continuously monitored for pseudoaneurysm expansion.

In conclusion, we successfully repaired a rare case of traumatic TR caused by PM rupture following cardiac contusion. This case highlights the importance of considering traumatic TR in patients with blunt cardiac trauma despite being a rare complication.

## Supplementary Information

Supplementary Material 1. Preoperative echocardiography reveals tricuspid valve prolapse caused by papillary muscle rupture, and a thin area of the right ventricular anterior wall. Color Doppler echocardiography detects severe tricuspid regurgitation and a bidirectional shunt across the atrial septum.

## Data Availability

Data sharing is not applicable to this article as no datasets were generated or analyzed during the current study.

## Abbreviations

TR: Tricuspid regurgitation
PM: Papillary muscle
RV: Right ventricular
ASP: Atrial septal perforation
